# Managing emerging infectious diseases: Is a federal system an impediment to effective laws?

**DOI:** 10.1186/1743-8462-1-7

**Published:** 2004-11-19

**Authors:** Genevieve Howse

**Affiliations:** 1 Centre for Public Health Law, School of Public Health, La Trobe University, Vic, Australia

## Abstract

In the 1980's and 1990's HIV/AIDS was the emerging infectious disease. In 2003–2004 we saw the emergence of SARS, Avian influenza and Anthrax in a man made form used for bioterrorism. Emergency powers legislation in Australia is a patchwork of Commonwealth quarantine laws and State and Territory based emergency powers in public health legislation. It is time for a review of such legislation and time for consideration of the efficacy of such legislation from a country wide perspective in an age when we have to consider the possibility of mass outbreaks of communicable diseases which ignore jurisdictional boundaries.

The management of infectious diseases in an increasingly complex world of mass international travel, globalization and terrorism heightens challenges for Federal, State and Territory Governments in ensuring that Australia's laws are sufficiently flexible to address the types of problems that may emerge.

In the 1980's and 1990's HIV/AIDS was the latest "emerging infectious disease". Considerable thought was put into the legislative response by a number of Australian jurisdictions. Particular attention had to be given to the unique features of the disease such as the method of transmission, the kinds of people who were at risk, and the protections needed by the community and the infected population to best manage the care of those infected and to minimize new infections. Health workers and researchers began to find that "the most effective strategies that we have so far found to help promote reduction of the spread of HIV involve the adoption of laws and policies which protect the rights of people most at risk of infection" [1]. A good example of a legislative response which adopts this approach is found in section 119 and 120 of the Victorian *Health Act *1958. These sections emphasize the need to protect the privacy of the infected individual and to undertake a staged response which is proportional to the risk presented by the infected individual. The legislation has been very effective with HIV and has been praised for its progressive approach [2].

In 2003 the community has been faced with the emergence of two new infectious diseases, SARS and Anthrax. Whilst there were no cases of either disease in Australia, the threat of a possible outbreak had to be acknowledged and a response planned. Anthrax is not a new infectious disease. Humans can become infected with anthrax by handling products from infected animals or by breathing in anthrax spores from infected animal products (like wool, for example). People also can become infected with gastrointestinal anthrax by eating undercooked meat from infected animals. However, its manufacture and use as a weapon for bioterrorism forces us to rethink its management in a new context.

These two infectious diseases have very different features from HIV which spreads only via transmission of infected bodily fluids such as blood or semen. SARS, by contrast is transmitted via droplets from infected cases which, as a result of coughing, carry the virus to close contacts [3] Thus, the infection profile of SARS requires planning for the possible overrun of Intensive Care Units and the likely infection of a number of ICU staff affecting both morale and capacity to cope. Anthrax raised different problems. These include the possible investigation of terrorist suspects alongside investigation of the outbreak of the infectious disease. Difficulties are also raised by likelihood of public panic, and the flooding of public health officials with reports of suspicious white powder.

In early 2004 the media reported the spread of avian influenza across South East Asia. This disease has different features from HIV/AIDS and SARS and an approach to an Australian outbreak would also be different. The main difference is in the source of transmission of the virus, that is, from infected birds to humans. There is very little difference [from ordinary influenza] in the symptoms (though these may vary in severity) or treatment of the virus [4] It is too early to predict whether this may be the next "emerging infectious disease", but its current spread has given rise to concern about such a possibility [5]

Australia is a federal system. There are two parallel sets of laws in operation. The Commonwealth Constitution sets out the legislative powers of the Commonwealth. Specific powers are listed in the Commonwealth constitution but State constitutions have broad powers covering matters such as peace, order and good governance. As the Commonwealth has no specific power to legislate with respect to health, other than the quarantine power, national legislative schemes in public health which rely upon a cooperative approach from all States and Territories are cumbersome and difficult.

Without a specific head of power, the Commonwealth has limited ability to legislate with respect to health. "That is, the legislative powers of the Commonwealth are specified in the Constitution and do not include expressly most of the activities that together comprise the field of public health"[6] For this reason, there are no Commonwealth emergency health powers except quarantine powers. Quarantine powers are currently restricted to isolation at the border of the country of people, plants, and animals to prevent the spread of disease. There is a real possibility that quarantine laws could have a broader scope. It depends on how widely the High Court would interpret section 51(x) of the Commonwealth Constitution. A quarantine law could override state laws as long as it remained a law "with respect to quarantine". However, "the power is potentially a colossus so far as the expansion of legislative authority in the fields of public health is concerned". [6]

The quarantine power would be the most likely candidate for a head of power on which to base development of commonwealth laws for the management of public health emergencies. Another possibility may be the external affairs power, if there was a relevant treaty or international agreement which could be given effect to in domestic law. However the legislation would have to be limited to laws giving effect to the treaty.

States and territories have a range of emergency powers available to them in their existing public health legislation. Some are relatively old. For example, the *Health Act *1911 (WA), *Public Health Act *1952 (NT) based on an 1898 Ordinance (Both these Acts are currently under review). Health emergency powers vary from one jurisdiction to another, but include powers to support disease surveillance, contact tracing and orders to restrict behavior or movement of individuals with an infectious disease in certain circumstances. There are also powers to recall food, search premises and seize property, close buildings and a range of other substantial and intrusive powers.

It is suggested that it is time to consider whether state and territory public health legislation contains sufficient measures to manage the outbreak of an infectious disease in a modern environment which includes mass travel, swift spread of infection and additional complexity raised by fears of bioterrorism.

Currently, in a public health emergency caused by the spread of an emerging infectious disease, Australia could need to rely on a patchwork of legislative measures to assist it to cope. Commonwealth quarantine laws and State and Territory powers in public health legislation may all be needed to address the problem. If an outbreak occurred on a border, or in some area where jurisdiction may be in doubt such as airspace or offshore and a state or territory response was required in addition to any quarantine measures, there could be confusion over jurisdiction for the application of State and Territory powers. State and Territory public health acts do not adequately provide for interjurisdictional communication and cooperation. There could also be difficulties if an infectious disease caused overseas deaths of people from more than one State or Territory in circumstances where an Australian coronial investigation was considered desirable. In such a situation, the jurisdiction of more than one Australian coroner would be triggered. Several State and Territory coronial laws could apply and there could be different inquests under different laws undertaken by different coroners into the same incident.

It is suggested that it is time to look at the efficiency of the emergency powers laws of Australia as a whole: to map the laws in each jurisdiction and the Commonwealth quarantine laws and to consider their effectiveness in the face of the outbreak of a fast moving, easily spread infectious disease. The efficacy of Australia's laws should also be considered in relation to bioterrorism. While there were no infections from anthrax in 2003 despite a great deal of media coverage and infections and deaths in the US, a responsible legislature ought to acknowledge the possibility and ensure that the law is ready to support a swift and effective response.

It is not enough to consider whether the individual pieces of legislation are up to the task of managing outbreaks of newly emerging infectious diseases. Indeed many of the jurisdictions are currently reviewing their public health legislation and will no doubt give proper consideration to this issue as part of the review. But who is thinking about how the legislation of all jurisdictions and the Commonwealth quarantine fits together? What powers enable communication and cooperation between jurisdictions about the outbreak of infectious disease? What kind of opportunity is there for a coordinated response? Can public health orders made in one jurisdiction travel to another jurisdiction when the infected individual travels? What arrangements can be made if an outbreak occurs on or close to a interstate border? What if there is an outbreak on a bus carrying passengers from Victoria, through South Australia to the Northern Territory?

It is encouraging to note that, even without specific legislation, there has been a mechanism to achieve communication and cooperation between jurisdictions through the Communicable Disease Network of Australia (CDNA). This Network has in fact been quite successful in fostering regular communication between the Communicable Disease Units across the country and has been involved in coordinated actions during a number of multistate outbreaks.

Despite the existence of this network and other good working relationships between government officials and various agencies in different jurisdictions, a serious outbreak of communicable disease would require the existence of legislative powers. Public health emergencies generate confusion, even panic. Clarity of powers and the way those powers interact with each other would be crucial in an emergency. It became apparent after the Bali tragedy in 2002 that coroner's jurisdiction was triggered differently in different jurisdictions and some acts did not support communication and cooperation when inquests might be needed for deaths of people ordinarily resident in several jurisdictions. The time to find the shortcomings in the legislation is well before the crisis.

A review of the efficacy of how these laws work together to protect the public health of all Australians should be undertaken. It has been possible to overcome the hangovers of federation for the betterment of all Australians in relation to corporations law. When doubts were recently raised about the constitutional basis of the corporations law scheme, the States and Territories were able to cooperate and refer the necessary powers to the Commonwealth to provide certainty about the laws which govern our corporations. Is our public health any less important than governance of our corporations? Could we cooperate to give ourselves certainty, flexibility and a consistent approach which protects the rights of those subject to some very broad powers?

The States and Territories are generally reluctant to refer powers to the Commonwealth. It may be time to seriously discuss referral of powers in the context of health emergency powers. At the very least, it is time that the Commonwealth, States and Territories recognised the need for the laws to work as a set of laws to protect the whole country, not simply individual laws to protect individual jurisdictions.

There has been work done internationally in this area. A model *State Emergency Health Powers Act *has been developed in the US in 2001 [7] In the preamble to this Act a rationale for its development is set out: "In the wake of the tragic events of September 11, 2001, our nation realizes that the Government's foremost responsibility is to protect the health, safety and wellbeing of its citizens. New and emerging dangers including emergent and resurgent infectious diseases and incidents of civilian mass casualties – pose serious and immediate threats to the population. A renewed focus on the prevention, detection, management and containment of public health emergencies is thus called for." The US, like Australia, is a Federal system. The model was intended to be taken up by those US states which wished to do so. To date, it has been passed in over half the US states. This bill would be an excellent starting point for development of an Australian model. There are a number of legislative mechanisms which could be used to support a nationally uniform approach to health emergency powers legislation in Australia.

The development and adoption of the model food legislation provides a useful model of a cooperative uniform approach. A model act was developed in consultation with all jurisdictions. It covered areas agreed to be core areas of the Act which ought to be the subject of a national approach and other provisions which were considered to be administrative and were to be adopted at the discretion of each jurisdiction. An intergovernmental agreement was signed as a mechanism to protect the uniformity of the legislation. The agreement sets up a Ministerial Council, supported by a Food Regulation Standing Committee. The Council has responsibility for deciding on proposals to amend the model [8] If a decision is made in favor of amendment, States and Territories will use their best endeavors to submit to their respective Parliaments, legislation which gives effect to the amendment.

The law is an important tool in supporting the management of the outbreak of infectious diseases. The existence of our Federal system has meant that we have a different approach in each State and Territory together with Commonwealth control of quarantine. Newly emerging infectious diseases creating real threats to public health in an era of easy mass travel, and the present threat of bioterrorism mean that it is time Australia examined all laws to contain and manage infectious disease outbreak. The laws should be examined both for their effectiveness in the areas they cover, and as part of a whole which ought enable a response which protects the health of all Australians, and crosses borders as easily as SARS or avian influenza.
